# Category Mistakes Electrified

**DOI:** 10.1007/s13164-023-00684-y

**Published:** 2023-07-10

**Authors:** Poppy Mankowitz

**Affiliations:** University of Bristol, Bristol, UK

## Abstract

Occurrences of sentences that are traditionally considered category mistakes, such as ‘The red number is divisible by three’, tend to elicit a sense of oddness in assessors. In attempting to explain this oddness, existing accounts in the philosophical literature commonly claim that occurrences of such sentences are associated with a defect or phenomenology unique to the class of category mistakes. It might be thought that recent work in experimental psycholinguistics—in particular, the recording of event-related brain potentials (patterns of voltage variation in the brain)—holds the potential to shed new light on this debate. I review the relevant experimental results, before arguing that they present advocates of accounts of category mistakes with a dilemma: either the uniqueness claims should be rejected, or the experimental technique in question cannot be used to test existing accounts of category mistakes in the manner that philosophers might hope.

## Introduction

Occurrences of sentences such as the following are often described within the philosophical literature as *category mistakes*:^1^

  (a): The red number is divisible by three.
  (b): Yasma is drinking the table.
  (c): The table is drinking water.

It is commonly claimed that such items belong to a class consisting of all and only the occurrences of sentences that are associated with a type of defect or phenomenology unique to genuine category mistakes.

If the uniqueness claims in general, or some account of category mistakes in particular, are substantive and falsifiable, then we should expect the potential for empirical testing. There is extensive discussion within the psycholinguistics literature of sentences that contain anomalous, incongruous or unexpected words. Hence we might anticipate fruitful results from incorporating empirical insights into the philosophical literature on category mistakes. To my knowledge, Elbourne (2016) is the first to pursue this illuminating approach. He reviews research on event-related brain potentials (patterns of voltage variation in the brain), before drawing several inferences relevant to adjudicating between accounts of category mistakes. I will argue that the psycholinguistics literature instead presents a dilemma: either the uniqueness claims should be rejected, since experimental results violate their empirical predictions; or existing accounts of category mistakes cannot be tested via the relevant experimental technique, in the manner that Elbourne and other theorists might hope.

In §(2), I characterise the uniqueness claims more clearly. In §(3), I provide an overview of the psycholinguistics literature on event-related brain potentials, and the N400 effect that is frequently elicited by words that are incongruous or unexpected. In §(4), I describe the dilemma that these results present for advocates of accounts of category mistakes.

## Accounts of Category Mistakes

§(2.1) sets out two uniqueness claims that are found in the philosophical literature on category mistakes, and explains their popularity. §(2.2) shows that a number of prominent accounts accept the uniqueness claims.

### The Uniqueness Claims

The following two claims are commonly accepted:

- There is a class to which ordinary occurrences of (1a)-(1c) belong consisting of *all and only* the occurrences of sentences that . . .
  - (*Unique Defect*) . . . are afflicted by a particular type of defect.
  - (*Unique Phenomenology*) . . . elicit a distinctive type of phenomenological state in assessors.

I will use the phrase ‘*candidate category mistakes*’ to refer to occurrences of sentences that are traditionally considered category mistakes, like ordinary occurrences of (1a)-(1c); and I will use the phrase ‘*genuine category mistakes*’ to refer to candidate category mistakes that belong to a class that satisfies one or both of the above uniqueness claims. This terminology allows sentences like (1a)-(1c) to be discussed without presupposing that they belong to a class characterised by a unique defect or phenomenology. For instance, those who reject the uniqueness claims might argue that some candidate category mistakes (e.g., (1a)) involve a different type of defect or phenomenology to other candidate category mistakes (e.g., (1c)), or that some candidate category mistakes share a defect or phenomenology with certain occurrences of sentences that are not candidate category mistakes. Advocates of Unique Defect identify a specific type of syntactic, semantic or pragmatic defect possessed by all and only genuine category mistakes. Advocates of Unique Phenomenology hold that the unique type of phenomenological state elicited by all and only genuine category mistakes is a particular ‘phenomenology of infelicity’ (Magidor 2013, p. 2) or sense of oddness that is phenomenologically distinct from other experiences of infelicity or oddness.

There are several reasons for the popularity of the uniqueness claims within the philosophical literature. First, those who are attempting to give an account of candidate category mistakes generally assume that they are targeting a unified phenomenon, because theorists with doubts about the existence of unifying features would be less likely to try to develop a uniform account. Second, an endorsement of Unique Defect is often implicitly or explicitly justified by the aim of theoretical simplicity, to be reassessed only if no uniform account of candidate category mistakes can be developed.^2^ Third, candidate category mistakes are frequently used to characterise some other linguistic phenomenon about which independent interest exists. For example, Lappin claims that the class of genuine category mistakes is co-extensive with the class of occurrences of sentences that are simultaneously syntactically well-formed and semantically ill-formed, meaning that ‘a theory of [genuine category mistakes] constitutes a theory of semantic ill-formedness in sentences’ (Lappin 1981, p. 2). Similarly, some accounts of metaphor hold that the presence of a genuine category mistake is what precludes the literal reading of an occurrence of a sentence and triggers a metaphorical reading (see Beardsley 1962), which suggests that an analysis of genuine category mistakes is essential to an account of non-literal meaning. If both uniqueness claims were to be rejected, then it would appear to follow that no quality—or at least, no linguistic defect or phenomenology—could be used to identify a class of genuine category mistakes; and it might then be argued that it is misguided to attempt to use such a class to characterise any other linguistic phenomenon. The linguistic phenomenon of independent interest would instead need to be characterised and analysed directly.

### Existing Accounts and the Uniqueness Claims

Following Magidor 2013, I distinguish accounts of candidate category mistakes based on the proposed source of their defect. *Semantic* accounts have been particularly popular (see Asher 2011; Drange 1966; Fodor and Katz 1963; Goddard and Routley 1973; Lappin 1981; Martin 1974; Ryle 1938; Thomason 1972; van Fraassen 1971). The only prominent advocate of a *syntactic* account is Chomsky (1965), and Magidor (2013) is the sole current advocate of a *pragmatic* account. This section shows that a broad range of existing accounts include commitment to Unique Defect and Unique Phenomenology.

Chomsky (1965) claims that genuine category mistakes involve a particular kind of syntactic defect. He takes the lexical entries for nouns to be associated with collections of *syntactic features* (1965, p. 82). For example, ‘number’ is marked as being not only a noun, but also a count noun and an abstract noun. The lexical entries for verbs and adjectives express *selectional restrictions* pertaining to certain features of their arguments (ibid., p. 95). For instance, the adjective ‘red’ requires its argument to be marked as non-abstract, hence the application of ‘red’ to the argument ‘number’ would involve a violation of this restriction. Chomsky’s account is committed to Unique Defect: there is a class of genuine category mistakes consisting of all and only those occurrences of sentences that involve the violation of selectional restrictions.^3^ Whether the account is committed to Unique Phenomenology is harder to establish. On one hand, he accepts that candidate category mistakes have a ‘special character’ (ibid., p. 151). On the other hand, he concedes that some sentences for which selectional restrictions are violated seem more intuitively deviant than others (see my discussion in fn. 3), which suggests that he attributes either a difference in kind or degree of phenomenological effect.

According to Lappin (1981), genuine category mistakes are semantically defective by virtue of lacking truth conditions, which causes them to be necessarily undefined. Lappin explicitly commits himself to Unique Defect, stating that an occurrence of a sentence is a genuine category mistake ‘if and only if it is syntactically well-formed and lacks truth-value in all possible worlds’ (1981, p. 67). Whether he accepts Unique Phenomenology is less clear. He holds that ‘speakers are capable of identifying paradigm cases of semantic well-formedness, and deviance’, which indicates that the property of being a genuine category mistake ‘is a property which speakers perceive’ (ibid., p. 3). Speakers’ perception of this property might be thought to constitute a distinctive phenomenological state, although Lappin does not discuss this issue.

Like Lappin, Asher (2011) holds that genuine category mistakes are characterised by their semantic ill-formedness. He develops a system where predicates encode presuppositions about the types of their arguments, such that ‘[i]f an argument in a predication cannot satisfy the type requirements of the predicate, then the predication cannot be interpreted and fails to result in a well formed logical form capable of having a truth value’ (2011, p. 7). Asher holds that a genuine category mistake is present if and only if a type inconsistency of a particular kind arises, namely one where ‘the type requirements of the predicate and argument give rise to incompatible individuation conditions’ (ibid., p. 50); hence he accepts Unique Defect. He indicates that individuals have ways of recognising genuine category mistakes: ‘Thinking about whether a competent speaker could entertain or believe the proposition expressed by a sentence gives us another means to distinguish between those sentences [that count as genuine category mistakes] and those that do not’ (ibid., p. 5). If competent speakers are unable to entertain or believe a proposition expressed by genuine category mistakes, then this might be taken to predict that they will experience a distinctive phenomenology when called upon to assess a candidate category mistake; although, like Chomsky and Lappin, Asher is not explicit on this issue. Other semantic accounts that explicitly commit themselves to Unique Defect include (Drange 1966; Goddard and Routley 1973; Martin 1974; Ryle 1938; Thomason 1972).^4^

Magidor’s (2013) pragmatic account begins with the notion of a *presupposition* as information that is required to already be present in the common ground of the relevant context. The absence of this information results in *presupposition failure*. While semantic notions of presuppositions (see Asher 2011) entail that presupposition failure leads to semantic undefinedness, Magidor holds that presupposition failure simply causes meaningful, valued occurrences of sentences to elicit a sense of pragmatic infelicity in assessors. Magidor argues that genuine category mistakes are cases where a presupposition triggered by a predicate fails.

Given that occurrences of non-candidate category mistakes might also involve presupposition failure, an advocate of this view need not commit herself to Unique Defect. However, Magidor appears to accept this thesis, holding that ‘there is a distinctive class of infelicitous sentences, ones that seem infelicitous in a similar manner to [(1a)-(1c)], [which] points to a linguistic phenomenon: the phenomenon of category mistakes’ (2013, pp. 1-2).^5^ She furthermore provides a particularly clear endorsement of Unique Phenomenology, claiming that ‘different category mistakes at least resemble each other by exhibiting a very similar phenomenology of infelicity (after all, it is precisely this distinctive phenomenology that has been used to characterize the relevant class of sentences)’ (ibid., p. 2). She emphasises that ‘the kind of infelicity that is associated with category mistakes seems very different in character than that associated with sentences which are otherwise trivially false or trivially true. An utterance of the sentence ‘London is in England and London is not in England’ may indeed be odd, but it is odd in very different manner than ‘Green ideas sleep furiously” (ibid., p. 113).

The current section indicates that an explicit commitment to Unique Defect is the dominant approach amongst developed accounts of category mistakes, whereas an explicit endorsement of Unique Phenomenology is rarer.^6^ However, a number of theorists who do not develop accounts of candidate category mistakes endorse Unique Phenomenology in passing. For instance, Shaw (2016, p. 213) states that candidate category mistakes ‘tend to sound infelicitous in a distinctive way’, and Szabó (2015, p. 289) holds that they ‘carry a peculiar sense of anomaly. We know them when we hear them (more or less)’. It is also worth noting that several theorists have proposed that an adequate analysis of candidate category mistakes must include a ‘general criterion for identifying’ genuine category mistakes (Lappin 1981, p. 15).^7^ Since such a criterion is possible only if a unique type of defect or phenomenology allows the identification of a class of genuine category mistakes, the proposal of this general adequacy condition reflects the extent to which the philosophical literature has taken the uniqueness claims for granted.

After describing an experimental technique widely used in psycholinguistics in §(3), the implications that it holds for accounts of category mistakes and the uniqueness claims will be considered in §(4).

## Anomalous Sentences in Psycholinguistics

Event-related brain potentials are a popular way to study real-time language processing. §(3.1) gives an overview of this technique. §(3.2) discusses the N400 effect, a type of component in event-related brain potentials that is often elicited by anomalous occurrences of sentences like candidate category mistakes.

### Event-related Brain Potentials

Neurons in the brain give off electrical fields, and the associated changes in voltage can be detected via electrodes placed on the scalp. An *event-related brain potential* (ERP) consists of the pattern of voltage variation that occurs during a period time-locked to some stimulus (Coles and Rugg 1996, pp. 1-2). An ERP is presented as a waveform with positive and negative peaks that occur at specified time-points. Potentially significant parts of the waveform are described as ‘ERP *components*’, and the same component is often identified in ERP data from multiple experiments. Components are typically individuated on the basis of the amplitude of their peaks, their latency (in milliseconds), the electrode locations at which they are detected most strongly, and the type of events to which they are sensitive.^8^ The names given to common ERP components often reflect their polarity and the latency of their peak relative to the relevant event. For example, the N400 component (see §(3.2)) is a *negative* wave that typically peaks *400ms* after the relevant event. I will describe an ERP component as *indexing* a particular type of cognitive process or state when that component reliably co-varies with that type of process or state under appropriate experimental conditions. The assumption that at least some components index types of cognitive processes and states is inherent in ERP research, even though this cannot be directly tested by recording brain activity.

The use of the ERP technique has a number of appealing features. First, by measuring voltage variation, ERPs capture neural processes in real-time, in contrast with techniques that measure blood flow or volume. Second, the technique measures the neural processes related to an event (say, the presentation of a sentence) without requiring participants to complete an additional task that might affect their response to that event (say, pushing a button to indicate their judgement of that sentence).

The technique also has several limitations. First, ERPs can only record the net electrical fields of large populations of neurons that are simultaneously active and arranged in a certain type of geometric configuration. Since many neural processes fail to meet these criteria, it follows that ‘there are almost certainly numerous functionally important neural processes that cannot be detected using the ERP technique’ (Coles and Rugg 1996, pp. 2-3). Second, a number of assumptions are required in order to draw inferences about the cognitive processes and states indexed by a particular ERP component. For example, two instances of ERP components might differ because they record distinct types of neural processes, or because they record the same type of neural process active to differing degrees. Researchers generally assume that differences in polarity or scalp distribution of ERP components indicate that they record qualitatively distinct neural processes, whereas differences in amplitude or (slight) differences in latency reflect variations in the degree or timing of a single type of neural process or cluster of interrelated neural processes (van Berkum 2004, p. 242). In order to explain the cognitive significance of the neural processes that they record, ERP researchers additionally tend to assume that multiple types of cognitive processes or states cannot be associated with the exact same type of neural process, and that different types of neural processes cannot give rise to the exact same type of cognitive process or state (Rugg and Coles 1996, p. 33). All of these assumptions are reasonable, whilst also being important in order to draw inferences about the significance of ERP components. Still, they are far from immune to doubt; although empirical data are unlikely to be relevant to their assessment.

The limitations of the ERP technique have not prevented its extensive use to study cognitive processes and states, including motor preparation (Kornhuber and Deecke 1965; Loveless and Sanford 1974; Walter et al. 1964), attention (Hillyard and Hansen 1986; Näätänen et al. 1978; Sutton et al. 1965), image processing (Barrett and Rugg 1990; Holcomb and McPherson 1994) and linguistic processing (see §(3.2)).

### The N400 Effect

Kutas and Hillyard (1980b) were the first to identify the *N400 component*, a negative wave beginning about 250ms and peaking around 400ms after the onset of a target event. In their study, participants read sentences presented one word at a time, where some sentences had an unexpected and incongruous final word. The unexpected final words elicited a notable N400 component relative to the expected ones (as in (2)).

2. He spread the warm bread with (butter / socks).

It was later shown that most words in a sentence elicit an N400 component (see Kutas et al. 1988; van Petten and Kutas 1990). Yet when a word has a semantic meaning that is incongruous or unexpected relative to the sentential or broader context, the amplitude of the N400 component is notably higher than for an identical sentence with an expected word. The phrase ‘N400 *effect*’ is used to describe the significantly higher amplitude of N400 components elicited by target words relative to the N400 components elicited by control words.

The N400 effect is typically not elicited by words that are physically anomalous (e.g., printed in a larger font; see Kutas and Hillyard 1980a) or syntactically anomalous (which often elicit the *P600 effect*, a positive wave that peaks 600ms after the relevant word; see Kaan et al. 2000; Neville et al. 1991; Osterhout and Holcomb 1992). An obvious inference would therefore be that the N400 effect is an ‘electrophysiological sign of the “reprocessing” of semantically anomalous information’ (Kutas and Hillyard 1980b, p. 203).^9^ Since ‘semantically anomalous’ is used by some in the linguistics and philosophy literature to refer to candidate or genuine category mistakes (e.g., Asher 2011; Fodor and Katz 1963; Shaw 2016), it might be tempting to then infer that the N400 effect is elicited by words that reveal the sentences in which they are situated to be candidate or genuine category mistakes. Yet I now review a number of studies that identify N400 effects for items that are not candidate category mistakes.

N400 effects can be elicited by words that are unexpected relative to the sentential or discourse context, even when those words do not produce semantically anomalous sentences or candidate category mistakes.^10^ Kutas and Hillyard (1984) showed that sentence-final words that were coherent but unexpected relative to the sentential context elicit a larger N400 than more expected ones (as in (3a)). The same effect was demonstrated by Federmeier and Kutas (1999) when a prior sentence established the expectation for a particular completion (as in (3b)), and by van Berkum et al. (1999, 2003) when a pair of prior sentences established the expectation (as in (3c)).

3.
  (a): The bill was due at the end of the (month / hour).
  (b): They wanted to make the hotel look more like a tropical resort. So along the driveway, they planted rows of (palms / pines / tulips).
  (c): As agreed upon, Jane was to wake her sister and her brother at five o’clock in the morning. But the sister had already washed herself, and the brother had even got dressed. Jane told the brother that he was exceptionally (quick / slow).

Van Berkum et al. (1999, p. 665) report that ‘the N400 effect elicited in discourse is indistinguishable from the standard N400 effect elicited by words that are anomalous given the “local” semantics of a single sentence’, in terms of shape, latency, scalp distribution and even amplitude.

Next, research has indicated that violations of world knowledge, independent of any context established within the experimental trials, may elicit N400 effects. Hagoort et al. (2004) carried out an experiment where participants read sentences with a target word that yielded either a true sentence, a false sentence or a candidate category mistake (as in (4a)). Dudschig et al. (2016) replicated this study with a few methodological modifications, such as retaining an identical sentence-final target word across all conditions in order to rule out any word-based effect (as in (4b)).

4.
  (a): The Dutch trains are (yellow / white / sour) and very crowded.
  (b): (Zebras / ladybirds / Journeys) are stripy.

Hagoort et al. (2004, p. 439) reported that an N400 effect was obtained with respect to target words that yielded either a false sentence or a candidate category mistake, and that the effect for both types of word ‘was identical in onset and peak latency and was very similar in amplitude and topographic distribution’; although the amplitude of the N400 effect was slightly greater for candidate category mistakes than for false sentences. Dudschig et al. (2016) report similar findings.^11^

Research has also shown that presenting a series of coherent sentences without an indication of the broader discourse topic can elicit N400 effects, despite the absence of any occurrences of sentences that are incoherent, false or candidate category mistakes. For instance, St. George and Mannes (1994) made use of paragraphs where each sentence was locally coherent, but the main theme of each paragraph could not be identified when it was presented without a title (e.g., ‘Procedure for washing clothes’). The authors found that the words in the untitled paragraphs elicited an N400 effect relative to the words in the titled paragraphs. Their results indicate that ‘a semantically anomalous word, or even a violation of context, is not necessary to produce the N400; it is also produced in response to a *lack* of context’ (St. George and Mannes 1994, p. 72). Finally, N400 effects may be elicited by isolated lexical items, despite the absence of any occurrences of sentences whatsoever. Bentin et al. (1985) presented a series of words where each one was followed by either a semantically related or unrelated word (e.g., ‘tulip’, (‘lilac’ / ‘rain’)). They found that the N400 component elicited by the semantically related words had a smaller amplitude than that of the N400 elicited by unrelated words.

The studies just reviewed demonstrate that the presence of a candidate category mistake is not a necessary condition for the eliciting of an N400 effect. Yet they also indicate that many candidate category mistakes elicit N400 effects (e.g., ‘Journeys are stripy’). It might therefore be thought that the presence of a candidate category mistake is a sufficient condition for the eliciting of an N400 effect (in appropriate experimental conditions). I now review a series of studies that fail to identify N400 effects for candidate category mistakes.

*Thematic role animacy violations* like (1c) (‘The table is drinking water’) comprise an important class of candidate category mistakes that have surprised ERP researchers by eliciting P600 rather than N400 effects (see Hoeks et al. 2004; Kim and Osterhout 2005; Kuperberg et al. 2003, 2006, 2007). Kuperberg et al. (2003) presented participants with sentences where verbs assigned the thematic role of Agent to inanimate noun phrases that were better suited to the role of Theme (as in (5a)). They also presented sentences that involved non-thematic role pragmatic violations (as in (5b)).

5.
  (a): For breakfast the eggs would only eat toast and jam.
  (b): For breakfast the boys would only bury toast and jam.
  (c): For breakfast the boys would eats toast and jam.

They expected both types of incongruous verb to elicit N400 effects, since detecting the incongruity requires semantic and pragmatic processing. Yet while the verbs in the pragmatic violations elicited the expected N400 effect, the verbs in the thematic role violations failed to elicit such an effect to a significant degree; instead, these verbs elicited a significantly greater P600 component than the verbs in the pragmatic violations.^12^ Kuperberg et al. 2006 subsequently found that the P600 elicited by thematic role animacy violations is similar in morphology, duration and scalp distribution to the P600 characteristic of morphosyntactic violations (e.g., (5c)), but smaller in amplitude. Kuperberg et al. (2003, p. 118) take these results to suggest that ‘upon encountering the verb (“eat”), participants might contemplate a ‘repair’ whereby the NP (“eggs”) is taken to be the theme of the verb (“eat”)’, which ‘requires a syntactic re-analysis, since the sentence, as presented, cannot convey this thematic role’. Similar results have been replicated by Kim and Osterhout (2005), Kuperberg et al. (2007) and Stroud (2009); although a number of different explanations of these data have been advanced (see Bornkessel-Schlesewsky and Schlesewsky 2008; Brouwer et al. 2012, 2017).

In light of the data discussed in this section, a number of positions on the types of cognitive processes indexed by the N400 component have attained support. The *retrieval* view holds that the N400 component reflects the retrieval of the lexical information associated with a word from memory (see Brouwer et al. 2012, 2017; van Berkum 2009; Kutas and Federmeier 2000). The *integration* view holds that it reflects the integration of the meaning of a word into a semantic interpretation or representation (Brown and Hagoort 1993; Chwilla et al. 1995; Hagoort et al. 2004; van Berkum et al. 1999, 2003). An increasingly popular *hybrid* view holds that the N400 component reflects both retrieval and integration, which are seen as interrelated but distinct cognitive processes (Baggio 2018; Nieuwland et al. 2020b; Pylkkänen and Marantz 2003). According to all of these views, the N400 effect indicates that the relevant cognitive processes are being taxed to a greater degree. Several theorists have recently argued that the N400 reflects processes other than retrieval or integration, such as probabilistic pre-activation of representations of upcoming words (DeLong et al. 2005), or updating of a probabilistic representation of a described event (Hodapp and Rabovsky 2021; Rabovsky et al. 2018); although Nieuwland et al. (2020a, 2018) question the robustness of some key evidence for routine pre-activation. According to these views, the N400 effect indicates that an incoming word was previously afforded a comparatively low probability. On the other hand, the P600 component that arises for some candidate category mistakes has typically been linked to syntactic processing (though for alternative proposals, see Bornkessel-Schlesewsky and Schlesewsky 2008; Brouwer et al. 2012, 2017). Accordingly, the P600 effect has been thought to reflect cognitive processes associated with costly syntactic processing (Osterhout et al. 1994), syntactic reanalysis (Friederici 1995), or syntactic integration difficulty (Kaan et al. 2000).

To summarise, a lexical item that is incongruous or unexpected for reasons independent of physical or syntactic anomaly generally seems to be necessary for the eliciting of an N400 effect.^13^ While these lexical items are often words that reveal an occurrence of a sentence to be a candidate category mistake, the presence of a candidate category mistake is neither necessary (see (3a)-(4b)) nor sufficient (see (5a)) for eliciting an N400 effect.

## A Dilemma for Advocates of the Uniqueness Claims

§(3) showed that different types of linguistic defects are known to elicit different types of ERP components, and that the N400 effect is often elicited by anomalous sentences. It would therefore be natural to expect ERP research to hold some relevance for the philosophical literature on category mistakes. To my knowledge, the only existing paper to connect the ERP literature to category mistakes in this way is Elbourne 2016. Elbourne provides an illuminating review of a number of the experiments discussed in §(3.2) (specifically: Hagoort et al. 2004; Nieuwland and van Berkum 2006; van Berkum et al. 1999, 2003). He then makes a series of claims about these results: they are easier to reconcile with the predictions of Magidor’s pragmatic account of category mistakes than with a certain type of semantic account, they support the view that candidate category mistakes are alike in kind to certain non-candidate category mistakes, and the phenomenological state elicited by candidate category mistakes is indexed by the N400 effect. The latter claim in particular might give the impression that ERP results can provide evidence in favour of the uniqueness claims. In the current section, I argue that advocates of accounts of category mistakes face a dilemma: either the ERP literature motivates the rejection of the uniqueness claims, or the ERP technique cannot be used to test them.

In §(4.1), I set out two further claims that advocates of the uniqueness claims are likely to endorse. It follows that advocates of the uniqueness claims predict the N400 effect to be elicited by all and only genuine category mistakes, a prediction that is difficult to reconcile with the ERP literature. In §(4.2), I argue that there are four options for upholding the uniqueness claims in light of ERP results, all of which have unappealing features. The most promising of these options is to reject the relevance of the ERP technique to evaluating the uniqueness claims. In §(4.3), I argue that ERP results are not even helpful for evaluating specific accounts of category mistakes.

### Empirical Difficulties

Here are two plausible claims:

- (*Unique Index*) If there is a type of defect or type of phenomenological state associated with all and only the items in a particular class, then: any ERP component that indexes the processing of this type of defect or the presence of this type of phenomenological state would be elicited by all and only the items in that class (under appropriate conditions).
- (*N400 Index*) If any currently identified ERP component indexes the processing of a type of defect or the presence of a type of phenomenological state associated with a class of occurrences of sentences to which certain candidate category mistakes belong, then it is the N400 effect.

The plausibility of the Unique Index claim is difficult to deny, for advocates and opponents of the uniqueness claims alike. It does not entail commitment to a type of defect or type of phenomenological state uniquely associated with any class of items. Even if such a type of defect or phenomenological state exists for a given class of items, the Unique Index claim does not entail commitment to an ERP index of the processing of this type of defect or the presence of this type of phenomenological state; for recall that there is no guarantee that any given cognitive process or state will correlate with a neural process that is detectable via the ERP technique. Finally, if such an index exists, the claim is compatible with circumstances where that ERP component fails to be elicited by an item in the relevant class, or is elicited by an item outside of that class: an explanation need only be given of why the experimental conditions are inappropriate.

The plausibility of the N400 Index claim is also difficult to deny. It does not entail commitment to the claim that the N400 effect *is* an index of the relevant cognitive states. It only entails that the N400 effect is the sole current candidate for such an index. §(3.2) provides extensive evidence for this view, insofar as the only component reliably elicited by the majority of candidate category mistakes is an N400 component with an amplitude significantly higher than that of the N400 component elicited by controls. Furthermore, the N400 Index claim involves no commitment to the existence of a class of genuine category mistakes, with a unique defect or phenomenology. The claim is compatible with the view that the N400 effect indexes cognitive states that are elicited by all and only the occurrences of sentences in a class that includes certain candidate category mistakes *and certain non-candidate category mistakes*.

Given the plausibility of the Unique Index and N400 Index claims, advocates of Unique Defect and Unique Phenomenology are likely to accept them. Moreover, advocates of both uniqueness claims think that the antecedent of the Unique Index claim holds with respect to a class of genuine category mistakes. Hence they would grant that any component that indexes the processing of the defect or the presence of the phenomenology unique to genuine category mistakes would be elicited by all and only genuine category mistakes (under appropriate conditions). Since they think that the class of genuine category mistakes is a class to which certain (if not all) candidate category mistakes belong, they will also understand the N400 Index claim to link this class to the N400 effect. It follows that advocates of the uniqueness claims are committed to the following position:

- (*Empirical Uniqueness*) If any currently identified ERP component indexes the processing of the type of defect or the presence of the type of phenomenological state associated with genuine category mistakes, then it is the N400 effect, and the N400 effect would be elicited by all and only genuine category mistakes (under appropriate conditions).

Problems now arise for the advocate of the uniqueness claims, since §(3.2) shows that it is not the case that the N400 effect is elicited by all and only candidate category mistakes. First, numerous experiments have identified N400 effects for words that are unexpected, incongruous or difficult to contextualise, despite the fact that these words do not produce candidate category mistakes. Second, thematic role animacy violations elicit P600 effects rather than N400 effects, despite the fact that occurrences of these sentences are normally considered candidate category mistakes. It follows that, if the N400 effect indexes the processing of a type of defect or the presence of a type of phenomenology associated with certain candidate category mistakes, then this type of cognitive process or state is not associated with all and only candidate category mistakes; and it would be difficult to see how this type of cognitive process or state could then be associated with all and only genuine category mistakes.

### The Dilemma

At this point, the advocate of the uniqueness claims has four options. The first option would be to reject Empirical Uniqueness. Given that this latter claim is entailed by the conjunction of the uniqueness claims with the Unique Index and N400 Index claims, at least one of these claims would need to be rejected. Yet §(4.1) argued that the Unique Index and N400 Index claims were independently plausible, for advocates and opponents of the uniqueness claims alike.

The three additional options are strategies for reconciling the ERP data with Empirical Uniqueness. The first strategy consists of arguing that the N400 effect *is* elicited by all and only *genuine* category mistakes, despite the fact that it is not elicited by all and only *candidate* category mistakes. In other words, occurrences of sentences such as (6a) (which failed to elicit an N400 effect in Kuperberg et al. 2003) are not genuine category mistakes, whereas occurrences of sentences such as (6b)-(6d) (which elicited N400 effects in specific settings in Federmeier and Kutas 1999; Hagoort et al. 2004; van Berkum et al. 1999) are genuine category mistakes:

6.
  (a): For breakfast the eggs would only eat toast and jam.
  (b): The Dutch trains are white.
  (c): So along the driveway, they planted rows of tulips.
  (d): Jane told the brother that he was exceptionally slow.

Existing accounts that identify a defect unique to genuine category mistakes are often committed to the view that this defect is exhibited by occurrences of sentences like (6a). For instance, Chomsky (1965) predicts that the selectional restrictions of the verb ‘eat’ will be violated if its argument is the inanimate noun ‘egg’. Similarly, the prediction emerges from Magidor (2013, pp. 145-6) that ‘*x* eats *y*’ presupposes that *x* is capable of eating, a presupposition that fails when *x* is an egg. An advocate of this argument who wishes to uphold Unique Defect faces the challenge of providing an account that avoids such predictions.

Another challenge for advocates of this argument is to explain why occurrences of sentences like (6a) have consistently been categorised as candidate category mistakes (e.g., see Lappin 1981, p. 1; Magidor 2013, p. 1). Such an explanation would be difficult to supply, since at least some of those who have classified thematic role violations as candidate category mistakes take themselves to detect the phenomenology characteristic of genuine category mistakes. For instance, Magidor (2013, p. 1) reports that an occurrence of ‘The theory of relativity is eating breakfast’ strikes ‘most English speakers as highly infelicitous, and infelicitous in a similar way [to other candidate category mistakes]’. As Magidor later notes, any theorist who claims that an occurrence of a sentence has been misclassified as a genuine category mistake, despite classifiers’ beliefs that they detect the distinctive phenomenology, ‘divorces the notion of a category mistake from the phenomenological quality that was used to characterise the phenomenon in the first place’ (ibid., p. 41).

An additional challenge for advocates of the first strategy is to explain why occurrences of sentences such as (6b)-(6d) are routinely classified as non-candidate category mistakes. Such an explanation would also be difficult to supply, since those who claim to detect a phenomenology unique to genuine category mistakes would presumably deny that this phenomenology is elicited to any non-zero degree by occurrences of sentences like (6b)-(6d). After all, Magidor (2013, p. 113) claims that the phenomenology elicited by genuine category mistakes is ‘very different in character’ to that triggered by trivially false sentences, and it is reasonable to suppose that she would take the same position with respect to empirically false sentences like (6b).

The second way for an advocate of the uniqueness claims to uphold Empirical Uniqueness would be to deny the propriety of the experimental conditions that elicit N400 effects for non-candidate category mistakes or fail to elicit N400 effects for candidate category mistakes. Since the relevant results have been replicated multiple times, it would be implausible to postulate errors in data collection or analysis. One version of this strategy might question the contribution of discourse context to the experimental results. Some accounts hold that an occurrence of a sentence may count as a genuine category mistake when presented without context or with a particular type of context, while failing to count as a genuine category mistake when presented relative to a different type of context.^14^ Advocates of such accounts might therefore claim that the appropriate conditions for eliciting the cognitive processes or states uniquely associated with genuine category mistakes are ones where isolated sentences are presented. The difficulty with this argument is that (6a) and (6b) were presented as isolated sentences, yet the first candidate category mistake failed to elicit N400 effects whereas the second non-candidate category mistake elicited such effects. While an advocate of the second strategy might identify other aspects of the experimental conditions that are inappropriate, there are no further obvious candidates.

In order to maintain that all and only genuine category elicit N400 effects (under appropriate conditions), one of the two strategies just described must be pursued. The third and final way for an advocate of the uniqueness claims to uphold Empirical Uniqueness would be to deny its antecedent. That is, it might be denied that any currently identified ERP component indexes the processing of the defect or the presence of the phenomenology unique to genuine category mistakes. To avoid accusations of an *ad hoc* reaction to empirically unfavourable results, an advocate of this strategy might appeal to the fact that some important neural processes lack the properties required for detection via the ERP technique (see §(3.1)). Indeed, there are independent reasons to doubt that any existing *or future* component would be uniquely elicited by a class of genuine category mistakes. ERP researchers tend to assume that the types of cognitive processes for which they record neural correlates are fairly general and coarse-grained (syntactic reanalysis, motor preparation, etc.). This assumption leads to two expectations. First, any ERP component will probably be elicited by multiple types of stimuli. In other words, ERP researchers will consider it unlikely that *any* ERP component indexes a cognitive process uniquely associated with *any* class of stimuli, unless the class is characterised in a highly general or circular way (e.g., so that it includes all and only stimuli that tax semantic integration processes). A second expectation is that, if there were to be a distinctive type of phenomenological state elicited by genuine category mistakes, then it would not be the sort of cognitive state with an ERP index, but it might result from many different types of cognitive processes that *do* have an ERP index.^15^ For instance, an individual could experience a subjectively indiscernible sense of infelicity whenever she encounters candidate category mistakes, despite the fact that she sometimes attempts a syntactic reanalysis, sometimes retrieves and integrates the meaning of a word with difficulty, and sometimes simply concludes that the sentence is uninterpretable. ERP data are more likely to provide evidence of these general cognitive processes than of some specific phenomenological state that can result from them. For ERP researchers, it follows straightforwardly from these expectations that no ERP components index cognitive processes or states elicited by all and only genuine category mistakes, independent of anything to do with category mistakes or the N400, and irrespective of whether the uniqueness claims hold.

The problems encountered by the first three options available to the advocate of the uniqueness claims render them difficult to defend. The fourth option appears to be the most promising. The advocate of Unique Defect or Unique Phenomenology therefore faces a dilemma: either she upholds the prediction that the N400 effect will be elicited by all and only genuine category mistakes while accepting that ERP data violate this prediction, or she denies that any currently identified (and, in all likelihood, future) ERP component indexes the processing of the defect or the presence of the phenomenology unique to genuine category mistakes. In the former case, there is evidence against the uniqueness claims. In the latter case, the uniqueness claims are not empirically testable via the ERP technique.

### Evaluating Particular Accounts of Category Mistakes

Suppose we grant that ERP results can provide no evidence in favour of the uniqueness claims. Can ERP results be used to evaluate particular accounts of category mistakes? I will briefly explain why they cannot easily be put to this use.

Elbourne (2016) contends that ERP data conform with the predictions of Magidor’s pragmatic account of category mistakes while violating the predictions of semantic accounts that classify candidate category mistakes as meaningful but lacking a binary truth value (e.g., Thomason 1972). He takes the first account to predict quicker recognition of multi-sentential candidate category mistakes (e.g., ‘The thing John just mentioned is green. The thing he mentioned is the number two’) than the second account, because the second account apparently predicts that an earlier sentence must be semantically reanalysed first. To assess these predictions, Elbourne (2016, p. 555) considers ‘relevantly similar’ multi-sentence discourses that have been employed in ERP experiments, focusing on those used in van Berkum et al. 1999, 2003 (e.g., (3c) in §(3.2)). Elbourne (p. 556) concludes that the N400 effect elicited in these experiments indicates that assessors are ‘capable of detecting discourse-level anomaly caused by a particular word before the speaker has even finished saying it, and no more slowly than they detect sentence-level anomaly’, in accordance with the predictions of a pragmatic account of category mistakes. The problem with this inference is that predictions about when assessors will detect a defect or phenomenological state associated with candidate category mistakes do not automatically entail predictions about the onset of N400 effects. That is, the detection of a defect or phenomenological state need not occur at the same time as the cognitive processes indexed by the N400 component.^16^

These observations illustrate a broader lesson. An advocate of an account of category mistakes must begin by endorsing a particular view of the cognitive processes indexed by the N400: retrieval, integration, updating of a probabilistic representation, and so on. Then she must explain the relation between the timing of the relevant cognitive processes and the detection of a candidate category mistake. She might also try to issue predictions about how particular stimuli will modulate the amplitude of N400 components. Only after completing these tasks could results pertaining to the N400 component be used to evaluate particular accounts of category mistakes. However, the ERP literature has reached no consensus on the cognitive significance of the N400 component, and existing accounts of category mistakes do not seem to offer any clear predictions about the cognitive effects of a word-by-word reading of candidate category mistakes.

Should advocates of particular accounts of category mistakes therefore give up hope of finding empirical support for their views? It would be a troubling outcome if these advocates were forced to defend accounts that could not be evaluated by any experimental methods. An alternative approach would be for philosophers to work more closely with cognitive scientists and psycholinguists, in order to identify testable predictions of particular accounts. Some of these predictions might be testable via the ERP technique, at least when a particular view of the cognitive processes indexed by certain ERP components is endorsed. Perhaps other predictions might be testable via alternative methods.

## Conclusion

Advocates of particular accounts of category mistakes, or of the general view that there is a class characterised by a unique defect or phenomenology containing all and only the genuine category mistakes, might hope that their position is amenable to empirical testing. Elbourne (2016) pursues the interesting strategy of using ERP results centering on N400 effects to draw inferences about the debate. He additionally thinks that ‘[t]he rich empirical literature on N400 effects will hopefully offer us further insights into this phenomenon [of category mistakes] in the future’ (Elbourne 2016, p. 557). Having reviewed the ERP literature, I argued that it cannot provide support for the uniqueness claims. Either it motivates the rejection of the uniqueness claims, or the ERP technique cannot be used to test them. Moreover, ERP results currently provide no help in evaluating existing accounts of category mistakes. In order to draw inferences from the ERP literature, philosophers would need to relate accounts of category mistakes to predictions about the activity of cognitive processes indexed by specific ERP components. While this task might be achievable in the future, it would require philosophers to work closely with cognitive scientists and psycholinguists.

## Data Availability

Not applicable

## Footnotes

1 I take an *occurrence* of a sentence to be a pairing of a sentence with a context of utterance. I focus on (1a)-(1c) as paradigm candidate category mistakes, due to their being particularly uncontroversial and simple examples. More complex examples that include quantifier expressions, connectives or multiple sentences have also been classified as category mistakes, such as occurrences of the following:

- Some number is green. (Magidor 2013, p. 56)
- John is in the library or prime numbers are hungry. (Lappin 1981, p. 132)
- The thing John just mentioned is green. The thing he mentioned is the number two. (Magidor 2016, p. 582)

2 For example, Magidor (2013, p. 2) writes: ‘I adopt as a working hypothesis the assumption that it is possible to give a uniform account of category mistakes, or in other words that the infelicity of different category mistakes arises for similar reasons. [. . .] Unless it turns out that no uniform account is ultimately successful, this hypothesis should be maintained’. Note that her uniformity hypothesis is the view that there is a particular defect exhibited by all, *but not necessarily only*, candidate category mistakes.

3 One complication is that Chomsky takes selectional restrictions to be violated in a sentence where an adjective or verb requiring a count noun combines with a non-count noun. If all violations of selectional restrictions produce genuine category mistakes, then occurrences of sentences like ‘Yan saw numerous dirt(s)’ should count as genuine category mistakes, even though they are generally not considered candidate category mistakes. Chomsky (1965, pp. 50-1) oberves: ‘It seems that sentences deviating from selectional rules that involve “higher-level” lexical features such as [Count] are much less acceptable and are more difficult to interpret than those that involve such “lower-level” features as [Human]’; but he also claims that the ‘special character’ of canonical examples of candidate category mistakes is ‘not attributable to the fact that these sentences violate rules involving “low-level features,” but rather to the fact that the rules that they violate are selectional rules’. This indicates that he would classify a sentence like ‘Yan saw numerous dirt(s)’ as a genuine category mistake, albeit one that might strike assessors as more deviant than other candidate category mistakes.

4 Ryle (1938, p. 200) holds that an occurrence of a sentence is a genuine category mistake when it ‘is (not true or false but) nonsensical or absurd, although its vocabulary is conventional and its grammatical construction is regular, [. . .] because at least one ingredient expression in it is not of the right type to be coupled or to be coupled in that way with the other ingredient expression or expressions in it’. However, Ryle (pp. 200-1) takes the class of genuine category mistakes to include some sentences not traditionally considered category mistakes, such as ‘I am now lying’. Drange (1966, p. 111) identifies the class of genuine category mistakes as all and only the occurrences of sentences that express ‘unthinkable propositions’, which result from attempts at combining a thing and a property for which we cannot ‘put the concept of that thing and the concept of that property together in thought’. Thomason (1972) claims that ‘a formula having the form **Pa** is to be sortally incorrect relative to a sortal specification if the referent of **a** does not belong to the sort assigned to **P**’ (p. 221), where ‘[a] sortal specification is a kind of partial valuation of a formal language’ (p. 222) that assigns to each predicate *P* of the language a subset of logical space ‘to be thought of as containing those points of which **P** can be affirmed or denied’ (p. 224). While the resulting set of genuine category mistakes only contains occurrences of sentences that can be translated into atomic formulas within a formal first-order language, Thomason (pp. 238-9) states that ‘the notion of sortal incorrectness does not extend very well to complex sentences’. Goddard and Routley (1973) attribute ‘significance ranges’ to expressions, before identifying a class of genuine category mistakes consisting of all and only grammatical occurrences of sentences that contain expressions with incompatible significance ranges. Finally, Martin (1975, p. 66) claims that ‘a category mistake is a failure of what we may call sortal presupposition’.

5 Note that Magidor (2013) explicitly commits herself solely to the view that *all* genuine category mistakes exhibit a particular defect (see my discussion in fn. 2); although she is additionally sympathetic to the view that *only* genuine category mistakes exhibit the relevant defect (p.c).

6 In fact, every existing account that I have encountered appears to commit itself to Unique Defect; although the current paper does not attempt to establish this.

7 Also see Drange (1966). Though note that some (e.g., Magidor 2013; Thomason 1972) have rejected the necessity or even possibility of providing such a criterion.

8 A pattern of voltage variation may count as an ERP component in this sense even if activity in multiple parts of the brain contribute to that pattern. For instance, the N400 component (see §(3.2)) is likely to reflect the activation of distinct neural generators (Nieuwland et al. 2020b). Note that the activity of multiple neural generators might still be thought of as contributing to a single neural process, although in some cases it will be better classified as contributing to multiple neural processes that are possibly related.

9 While this is an obvious inference to draw based on the earlier studies, it is not an inference that recent theorists tend to make, due in part to the results reviewed below. I return to contemporary views of the cognitive processes indexed by the N400 component at the end of the current section.

10 The fact that the described experiments present sentences that are incongruous relative to preceding sentences need not preclude the presence of candidate category mistakes: for Elbourne (2016) claims that there are multi-sentential candidate category mistakes (e.g., (c) in fn. 1), and Goldwater (2018) argues that Ryle (1938, 1949) originally conceived of category mistakes as conjoined sentences where neither conjunct is required to be a candidate category mistake. Still, none of the relevant stimuli in the described experiments count as single-sentence or multi-sentential candidate category mistakes.

11 Dudschig et al. identified a slightly earlier onset of N400 effects for candidate category mistakes than for false sentences when they used a ‘new exploratory method’ of temporal analysis (2016, p. 44). They urged caution in interpreting these results, since traditional methods revealed no timing differences.

12 One might wonder whether target words in thematic role animacy violations *do* elicit the cognitive processes that produce N400 effects, but the partially overlapping P600 effect cancels out the appearance of N400 effects (I thank an anonymous reviewer for raising this point). Kuperberg et al. (2003, p. 126) consider and reject this idea, on the grounds that the difference in onset and peak latencies for N400 and P600 effects ‘would predict that P600 / N400 interactions would be largest towards the end of the 300-500-ms epoch and that the N400 effect elicited by the thematic role animacy violations would reach significance at the beginning of this epoch. In fact, there were no significant differences in amplitude between the waveform elicited by thematic role animacy violated verbs and non-violated verbs amplitude at the beginning of this epoch (300-350 ms)’. Kuperberg et al. (2007, fn. 7) further note that sentences that involve both syntactic violations and semantic anomaly elicit N400 and P600 effects (Hagoort 2003; Osterhout and Nicol 1999), hence these two components ‘do not simply cancel out one another’. The idea that the P600 elicited by thematic animacy violations obscures an N400 effect could only be maintained by holding that the former component has a much earlier onset than the P600 elicited by morphosyntactic violations (Kuperberg et al. 2007, fn. 7); yet there is currently no empirical or theoretical justification for this assumption, and it is difficult to see how it could be reconciled with the findings in Kuperberg et al. (2006) that the P600 elicited by thematic role animacy violations has a similar duration to the P600 elicited by morphosyntactic violations.

13 Some researchers claim to have identified N400 effects for incongruous images (see Barrett and Rugg 1990; Holcomb and McPherson 1994; Nigam et al. 1992; Willems et al. 2008). Though others have argued that the N400-like components elicited by lexical and non-lexical items record non-identical types of neural activity, insofar as they ‘reflect similar cortical computations occurring in different, but overlapping, populations of neurons’ (Kutas et al. 2006, p. 669).

14 For example, Magidor (2013, p. 151) claims that certain occurrences of sentences ‘can exhibit the relevant phenomenology relative to some speakers, while seeming felicitous to other speakers (ones who possess different background information, beliefs or assumptions)’.

15 I am grateful to an anonymous reviewer for emphasising this point.

16 Elbourne makes two other key claims. First, he states ‘[t]he impression we are left with from the neurolinguistics literature is that category mistakes and sentences that are implausible in other ways fall on a continuum and that the neurological reactions to them are basically the same in kind, differing only [...] in degree’ (2016, p. 554). This claim derives from the idea that N400 effects emerge for all but not only candidate category mistakes. However, as discussed in §(3.2) and §(4.2), thematic role animacy violations fail to elicit N400 effects but are typically considered to be candidate category mistakes. Elbourne’s final claim is that ‘[i]n the light of the neurolinguistics literature reviewed above, we can also conclude that category mistake phenomenology is an N400 response’ (p. 557). Given his awareness that some non-candidate category mistakes elicit N400 effects, he presumably takes ‘category mistake phenomenology’ to be a state elicited by some candidate category mistakes and some non-candidate category mistakes, such that advocates of Unique Phenomenology incorrectly treat this state as uniquely characteristic of genuine category mistakes. The trouble is that, as explained at the end of §(4.2), ERP researchers think that the N400 component indexes general cognitive processes (say, retrieval) rather than a phenomenological state. Moreover, the ERP literature provides no grounds for inferring that taxation of these general cognitive processes is identical with a distinctive sense of infelicity singled out by advocates of Unique Phenomenology.
